# Inhaled treatment adherence patterns and related barriers across three monthly follow-up waves among patients with cough variant asthma: a clinical natural language processing study

**DOI:** 10.3389/fimmu.2026.1966303

**Published:** 2026-10-05

**Authors:** Chun-juan Zhang, Qiu-yue Shen, Xiu-juan Ma, Min-feng Zhou

**Affiliations:** 1 Affiliated Haiyan Hospital of Jiaxing University, Haiyan People’s Hospital, Jiaxing, Zhejiang, China; 2 School of Public Health, Zhejiang University School of Medicine, Hangzhou, Zhejiang, China

**Keywords:** clinical natural language processing, cough variant asthma, generalized estimating equations, inhaled therapy, longitudinal study, medication adherence

## Abstract

**Objective:**

To characterize adherence and adherence-related text features across three monthly telephone follow-ups among adults with cough variant asthma (CVA) newly initiating maintenance inhaled therapy and to evaluate their contemporaneous associations.

**Methods:**

This single-center, three-wave observational cohort classified adherence during the preceding month as high or low based on reported treatment implementation; records with insufficient information were unevaluable. A manually validated, rule-enhanced clinical natural language processing approach extracted seven non-mutually exclusive text features, including four prespecified patient-perceived barriers. Logistic generalized estimating equations (GEE) estimated population-averaged contemporaneous associations with high adherence. Complete-case GEE and wave-specific Firth models were conducted as sensitivity analyses.

**Results:**

Among 767 patients, adherence was evaluable for 732, 697, and 602 at Waves 1, 2, and 3, respectively; high adherence was documented in 94.4%, 73.9%, and 47.0% of evaluable records. Intentional discontinuation or dose reduction increased from 4.8% to 26.5%. The primary GEE included 2,031 observations. Lower odds of high adherence were associated with access or financial barriers (OR 0.28, 95% CI 0.16–0.49; P<0.001), illness- or treatment-related beliefs (OR 0.19, 95% CI 0.06–0.63; P = 0.007), and side-effect concerns or poor tolerability (OR 0.43, 95% CI 0.19–0.96; P = 0.040). Similar associations for access or financial barriers and illness- or treatment-related beliefs were observed in the complete-case and Wave 3 Firth analyses.

**Conclusions:**

High adherence was documented less frequently and patient-initiated treatment reduction more frequently at later waves. Access or financial barriers showed the most consistent contemporaneous association with lower odds of high adherence, whereas associations with illness- or treatment-related beliefs and side-effect concerns were less consistent across analyses. These record-based observational findings require prospective confirmation.

## 1 Introduction

Cough variant asthma (CVA) is an asthma phenotype in which persistent cough is the predominant or sole symptom. Wheeze and dyspnea may be absent, although airway hyperresponsiveness, variable airflow limitation, and airway inflammation may still be present (1–3). Inhaled corticosteroid (ICS)-containing therapy is central to management, but its effectiveness also depends on whether patients implement the prescribed regimen over time.

Medication adherence includes initiation, implementation, and persistence, which may differ within the same treatment course (4). In asthma, suboptimal adherence to ICS-containing therapy is common and has been associated with poorer clinical outcomes (5, 6). Treatment beliefs, access or financial barriers, and inhaler-use errors are among the factors associated with implementation problems (7, 8). A single assessment may therefore provide an incomplete account of medication-taking behavior during early treatment.

Routine telephone follow-up notes may capture missed doses, patient-initiated treatment changes, device difficulties, and reported reasons for nonadherence. These data are clinically informative but difficult to analyze because expressions vary across records. Clinical NLP can convert narrative information into structured variables, and rule-based methods offer transparent, auditable classification (9, 10). Previous asthma studies have commonly assessed adherence using self-report, clinician assessment, refill data, or electronic monitoring (11, 12). Evidence from repeated routine follow-up notes in CVA remains limited. We therefore described wave-specific adherence and related text features across three monthly follow-ups and evaluated their contemporaneous associations.

## 2 Materials and methods

### 2.1 Study design and setting

This single-center, real-world longitudinal observational cohort used routine management records from the respiratory outpatient clinic of Haiyan People’s Hospital. Consecutively eligible adults who newly initiated maintenance inhaled therapy between January 1, 2025, and May 30, 2026, were included. Three telephone follow-ups were scheduled approximately one month apart. Later-wave contact attempts were made irrespective of whether adherence was evaluable at the preceding wave. Each scheduled follow-up sought to assess inhaler use, symptoms, difficulties, and treatment changes; completed contacts with available documentation generated narrative clinical notes (**Figure 1**).

**Figure 1:**
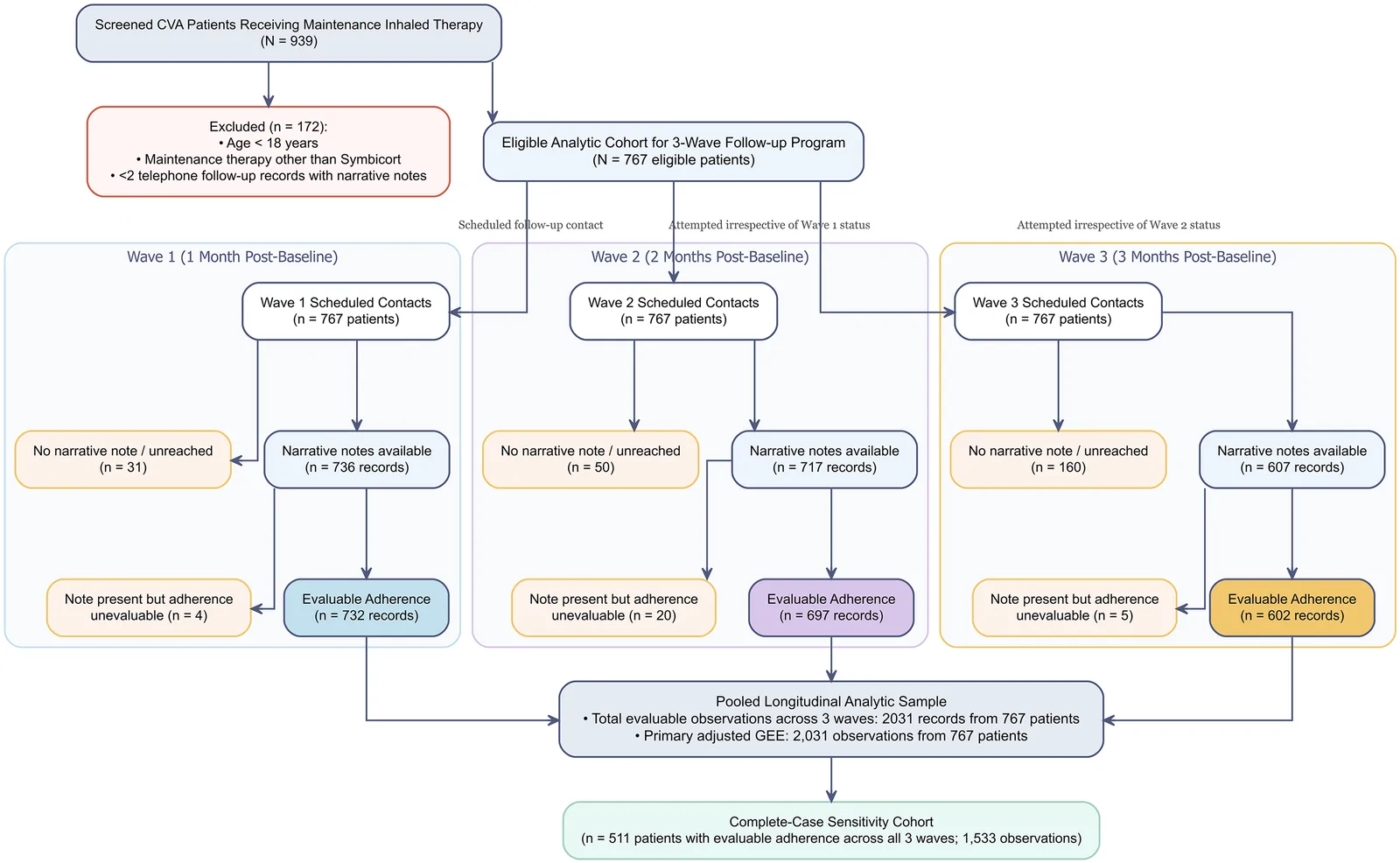
Flow diagram of cohort screening, scheduled follow-up contacts, narrative-note availability, adherence evaluability, and analytic samples. The complete three-wave sensitivity cohort comprised 511 participants and 1,533 observations.

The Ethics Committee of Haiyan People’s Hospital approved the study (approval no. 2024-33) and waived informed consent for this retrospective analysis of routine clinical records. The Haiyan County Health Bureau funded the study (grant no. 2025wyb006). Reporting followed the STROBE and RECORD statements (13, 14).

### 2.2 Participants and definition of CVA

Eligible participants were adults aged ≥18 years with CVA diagnosed during routine care by respiratory physicians with at least 15 years of clinical experience, who newly initiated maintenance inhaled therapy and had at least two scheduled telephone follow-up records with narrative documentation. Diagnosis was based on cough lasting >2 months as the sole or predominant respiratory symptom, integrated clinical assessment, and consideration of other common causes of chronic cough. Spirometry, bronchodilator reversibility testing, bronchial provocation testing, including methacholine challenge when performed, and FeNO were available only for a subset. Objective testing was not mandatory for cohort inclusion, and FeNO, when available, was considered supportive rather than diagnostic in isolation. Because uniform numerical results and testing thresholds were not available, diagnoses were not retrospectively re-adjudicated using a single test-based algorithm, and treatment response was not used as an eligibility criterion or for retrospective diagnostic confirmation.

Exclusion criteria were typical asthma with recurrent wheeze, chronic obstructive pulmonary disease, bronchiectasis, another major chronic respiratory disease likely to affect adherence assessment, use of a maintenance inhaler other than twice-daily Symbicort (budesonide/formoterol), or fewer than two scheduled follow-up records with narrative documentation. Insufficient information at an individual wave resulted in unevaluable adherence for that wave rather than participant exclusion. All eligible patients identified during the prespecified study period were included without a hypothesis-driven sample-size calculation.

### 2.3 Telephone follow-up and adherence outcome

Trained staff conducted routine semi-structured telephone follow-up using a clinical prompt guide rather than a validated adherence questionnaire. The follow-up addressed respondent identity, current respiratory symptoms, actual inhaler use during the preceding month, missed or irregular use, patient-initiated dose reduction or discontinuation, inhaler-device difficulties, tolerability concerns, medication access, alternative treatments, clinician-directed changes, and follow-up plans. The wording and order of prompts could vary according to the clinical context, and the information obtained was summarized in narrative clinical notes. The domains covered during follow-up are provided in Supplementary Appendix S1. High adherence was defined as no explicit documentation of recurrent or irregular missed use, patient-initiated dose reduction, or patient-initiated discontinuation during the preceding month. Explicit documentation of any of these behaviors was classified as low adherence. Clinician-directed treatment changes were excluded, and records with insufficient information were classified as unevaluable. This outcome reflected patient- or caregiver-reported treatment implementation and was not equivalent to prescription refill rates or electronic inhaler monitoring. All 767 participants newly initiated Symbicort (budesonide/formoterol) at cohort entry. Of these, 331 (43.2%) received the 160 strength and 436 (56.8%) received the 320 strength.

### 2.4 Clinical NLP identification of adherence-related text features

After text standardization, whitespace processing, and removal of uninformative content, we developed a rule-enhanced clinical NLP pipeline using keyword dictionaries, regular expressions, negation detection, and contextual exclusions. Seven non-mutually exclusive text features were identified: intentional discontinuation or dose reduction; forgetfulness or irregular use; side-effect concerns or poor tolerability; inhaler device-related difficulty; illness- or treatment-related beliefs; access or financial barriers; and alternative-therapy substitution. The last feature was positive only when the documented preference led to replacement, postponement, or reduction of prescribed inhaled treatment. Side-effect concerns or poor tolerability, inhaler device-related difficulty, illness- or treatment-related beliefs, and access or financial barriers were prespecified as patient-perceived barriers. The remaining features represented nonadherent behavior or treatment substitution and were included only in the extended co-documented analysis.

Negation rules excluded statements such as ‘did not stop treatment’ and ‘no side effects.’ Contextual exclusions removed clinician-directed treatment changes, recommendations, educational or hypothetical statements, and previous behaviors documented as resolved. Preference for traditional medicine, oral medication, or another approach was not sufficient by itself; concomitant use without an effect on prescribed inhaled-treatment implementation was excluded. Multiple features could be assigned to one record. A record could also be classified as low adherence without a specific feature when the note described nonadherent implementation but did not state a reason. **Supplementary Figure S1** summarizes the workflow, and **Supplementary Table S1** provides the operational definitions.

For internal validation, 200 follow-up records were randomly sampled. Two reviewers independently annotated all records while blinded to the rule-based classifications, and a third reviewer adjudicated disagreements. Consensus labels formed the reference standard. For intentional discontinuation or dose reduction, forgetfulness or irregular use, side-effect concerns or poor tolerability, inhaler device-related difficulty, illness- or treatment-related beliefs, access or financial barriers, and alternative-therapy substitution, sensitivity was 98.1%, 82.8%, 92.1%, 85.7%, 89.3%, 88.2%, and 87.9%, respectively. Corresponding specificity was 95.3%, 99.4%, 99.4%, 99.5%, 91.9%, 98.8%, and 97.0%; F1 scores were 0.927, 0.889, 0.946, 0.889, 0.746, 0.909, and 0.866; and Cohen’s kappa was 0.900, 0.872, 0.934, 0.881, 0.697, 0.891, and 0.839.

### 2.5 Longitudinal data structure and variable definitions

Data from the three follow-ups were converted to long format, allowing each patient to contribute multiple observations. Variables included patient identifier, follow-up wave, age, sex, contemporaneous adherence status, and the seven contemporaneous text features. Patient identifier was used as the clustering unit to account for within-patient correlation. Absence of a documented feature indicated only that it was not identified in the note; it did not establish absence of the barrier on standardized assessment.

### 2.6 Statistical analysis

Continuous variables are presented as mean ± standard deviation, and categorical variables as number and percentage. Descriptive comparisons used the Wilcoxon rank-sum, chi-square, or Fisher exact test, as appropriate. The primary analysis used binary logistic GEE with high adherence as the outcome, patient as the clustering unit, robust standard errors and wave as a categorical variable (15). Model 1 included four prespecified patient-perceived barriers: side-effect concerns or poor tolerability, inhaler device-related difficulty, illness- or treatment-related beliefs, and access or financial barriers. Model 2 additionally included intentional discontinuation or dose reduction, forgetfulness or irregular use, and alternative-therapy substitution. Both models adjusted for age per one-standard-deviation increase, sex, and wave. Because adherence and text features were usually derived from the same note, coefficients were interpreted as population-averaged contemporaneous associations. Features overlapping with the low-adherence definition were not interpreted as independent risk factors. Age- and sex-stratified analyses were exploratory. To explore whether these associations varied over time, we conducted two *post hoc* sensitivity analyses. First, multivariable Firth penalized logistic regression models were fitted separately at each wave because some barrier-by-outcome combinations were sparse. Each wave-specific model included the four patient-perceived barriers and adjusted for age, sex, and baseline Symbicort strength. Second, barrier-by-wave interaction terms were evaluated within the longitudinal GEE framework. Wave 1 was used as the reference, and the overall interaction P value was obtained by jointly testing the interaction terms for Waves 2 and 3. When an interaction could not be reliably estimated because of sparse records, it was reported as not estimable. The wave-specific and interaction analyses were considered exploratory (16). Subgroup analyses were stratified by age (<60 vs >=60 years) and sex within the four-barrier model, with between-group differences assessed using interaction terms.

Adherence evaluability at each wave and participant-level missingness were characterized. Age and sex were compared between evaluable and unevaluable records at each wave and between participants with complete versus any missing adherence. The seven evaluability patterns were grouped as complete, temporary, intermittent or recurrent, and persistent or terminal missingness. A logistic GEE modeled evaluability using age, sex, and wave. Prior-wave high-adherence proportions were also compared according to evaluability at the subsequent wave. Unevaluable adherence outcomes were not imputed.

Two analyses addressed treatment regimen and respondent source. First, the primary GEE was additionally adjusted for baseline Symbicort strength (320 versus 160). Second, respondent source was classified retrospectively from explicit narrative cues as patient source, caregiver source, or no narrative note. No separate explicit joint-interview category was identified. Because source classification was rule based and was not manually validated, it was treated as an audit variable rather than a validated exposure. High adherence was summarized by source, respondent-source GEE models compared caregiver- with patient-source records, and the primary model was repeated in patient-source records only. Analyses used R version 4.3.3. Tests were two-sided; P<0.05 was used as a descriptive threshold alongside effect estimates and 95% confidence intervals.

## 3 Results

### 3.1 Baseline characteristics

The analytic cohort comprised 767 patients who newly initiated maintenance budesonide/formoterol. At Wave 1, 736 participants had a narrative note and adherence was evaluable for 732. Among evaluable records, 691 (94.4%) were classified as high adherence and 41 (5.6%) as low adherence. Mean age was 46.0 ± 13.9 years, and 449 (61.3%) were women. Age and sex did not differ statistically between adherence groups. Intentional discontinuation or dose reduction, forgetfulness or irregular use, and side-effect concerns or poor tolerability were documented in 46.3%, 14.6%, and 17.1% of low-adherence records, compared with 2.3%, 0.3%, and 1.7% of high-adherence records (**Table 1**).

**Table 1:** Baseline characteristics according to adherence status at Wave 1.

Characteristic	Overall	High adherence (n=691)	Low adherence (n=41)	P value
Age, years	46.0 ± 13.9	46.1 ± 14.0	44.1 ± 12.7	0.348
Female sex	449 (61.3%)	421 (60.9%)	28 (68.3%)	0.411
Intentional discontinuation or dose reduction	35 (4.8%)	16 (2.3%)	19 (46.3%)	<0.001
Forgetfulness or irregular use	8 (1.1%)	2 (0.3%)	6 (14.6%)	<0.001
Side-effect concerns or poor tolerability	19 (2.6%)	12 (1.7%)	7 (17.1%)	<0.001
Inhaler device-related difficulty	8 (1.1%)	6 (0.9%)	2 (4.9%)	0.069
Illness- or treatment-related beliefs	1 (0.1%)	0 (0.0%)	1 (2.4%)	0.056
Access or financial barriers	12 (1.6%)	10 (1.4%)	2 (4.9%)	0.142
Alternative-therapy substitution	12 (1.6%)	11 (1.6%)	1 (2.4%)	0.502

Values are mean ± standard deviation or n (%). P values are from descriptive comparisons and were not adjusted for multiple testing.

### 3.2 Cross-sectional and longitudinal distributions of adherence and related text features

Adherence was evaluable for 732, 697, and 602 participants at Waves 1, 2, and 3, respectively. High adherence was recorded in 94.4% (691/732), 73.9% (515/697), and 47.0% (283/602) of evaluable records; the corresponding low-adherence proportions were 5.6%, 26.1%, and 53.0% (**Figure 2A**; **Table 2**). These wave-specific proportions use only evaluable records and do not quantify individual-level deterioration. Adjacent-wave transitions are shown in **Figure 2B**, **Table 3**, and **Supplementary Figure S2**.

**Figure 2:**
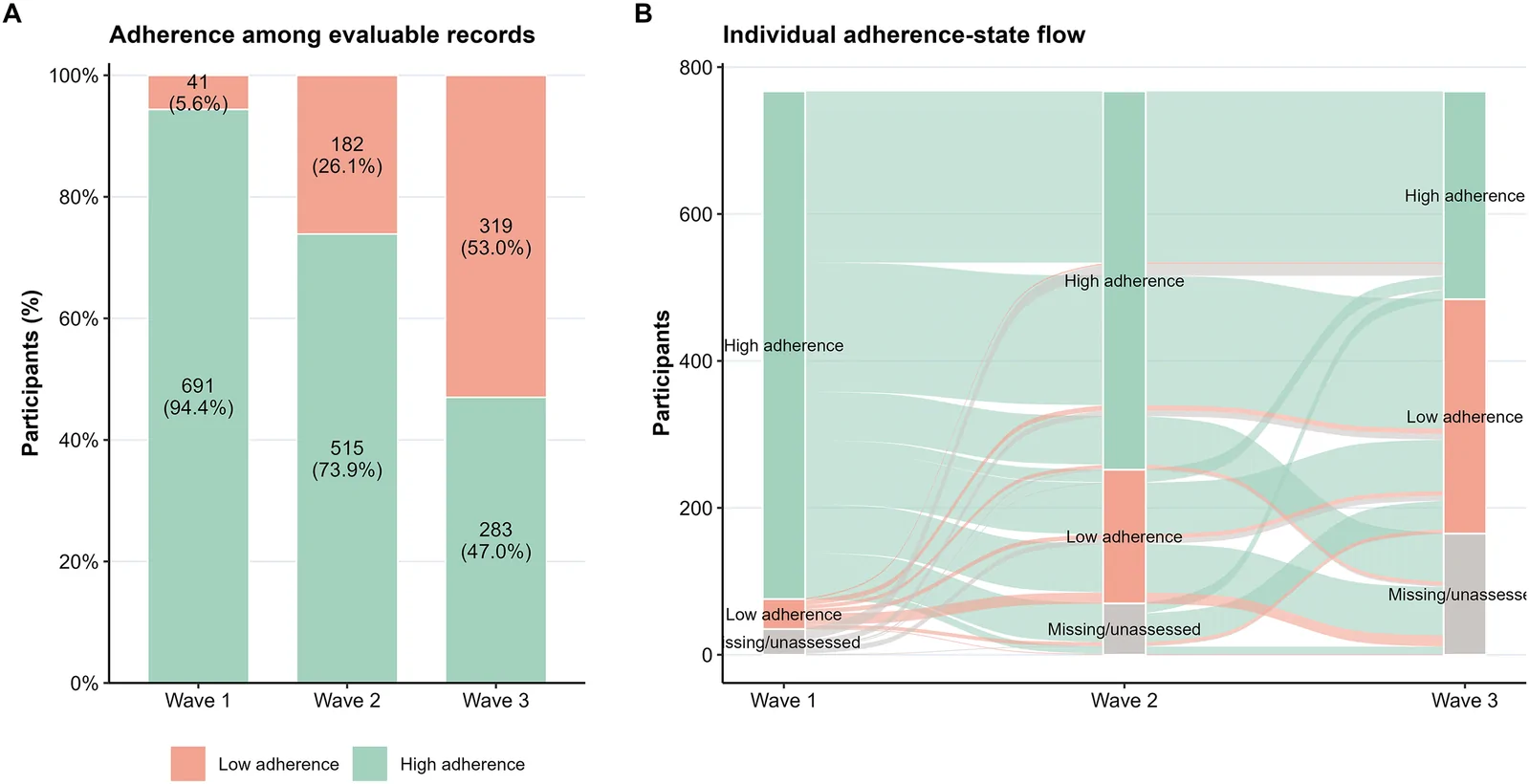
Adherence distribution and individual adherence-state flow. **(A)** High- and low-adherence distributions among evaluable records at each wave. **(B)** Flow among high adherence, low adherence, and missing or unevaluable status across the three waves. Numerical paired transitions are reported in **Table 3** and **Supplementary Figure S2**.

**Table 2:** Adherence and documented frequencies of seven text features across the three follow-up waves.

Measure	Wave 1, n (%)	Wave 2, n (%)	Wave 3, n (%)
Evaluable adherence	732 (95.4)	697 (90.9)	602 (78.5)
High adherence	691 (94.4)	515 (73.9)	283 (47.0)
Low adherence	41 (5.6)	182 (26.1)	319 (53.0)
Analyzable text	736 (96.0)	717 (93.5)	607 (79.1)
Access or financial barriers	12 (1.6)	30 (4.2)	30 (4.9)
Alternative-therapy substitution	14 (1.9)	35 (4.9)	17 (2.8)
Illness- or treatment-related beliefs	1 (0.1)	8 (1.1)	9 (1.5)
Forgetfulness or irregular use	8 (1.1)	31 (4.3)	18 (3.0)
Side-effect concerns or poor tolerability	19 (2.6)	19 (2.6)	10 (1.6)
Intentional discontinuation or dose reduction	35 (4.8)	107 (14.9)	161 (26.5)
Inhaler device-related difficulty	8 (1.1)	7 (1.0)	4 (0.7)

Text-feature denominators are records included in the NLP text analysis at each wave. Adherence percentages in the text use records with evaluable adherence as the denominator.

**Table 3:** Transitions in adherence state between adjacent follow-up waves.

Transition	From status	To status	n (%)
Wave 1 to Wave 2	Low adherence	Low adherence	21 (60.00%)
Wave 1 to Wave 2	Low adherence	High adherence	14 (40.00%)
Wave 1 to Wave 2	High adherence	Low adherence	153 (24.36%)
Wave 1 to Wave 2	High adherence	High adherence	475 (75.64%)
Wave 2 to Wave 3	Low adherence	Low adherence	83 (82.18%)
Wave 2 to Wave 3	Low adherence	High adherence	18 (17.82%)
Wave 2 to Wave 3	High adherence	Low adherence	191 (43.21%)
Wave 2 to Wave 3	High adherence	High adherence	251 (56.79%)

At least one positive record occurred for intentional discontinuation or dose reduction in 262/767 participants (34.2%), access or financial barriers in 66/767 (8.6%), alternative-therapy substitution in 55/767 (7.2%), forgetfulness or irregular use in 51/767 (6.6%), side-effect concerns or poor tolerability in 42/767 (5.5%), inhaler device-related difficulty in 19/767 (2.5%), and illness- or treatment-related beliefs in 18/767 (2.3%) (**Supplementary Figure S3**). Across the three waves, intentional discontinuation or dose reduction was documented in 4.8%, 14.9%, and 26.5% of analyzable text records. Wave-specific documented frequencies for the other features are shown in **Figure 3** and **Table 2**. **Supplementary Figures S4**–**S6** present wave-specific heatmaps, adjacent-wave documented dynamics, and pairwise correlations.

**Figure 3:**
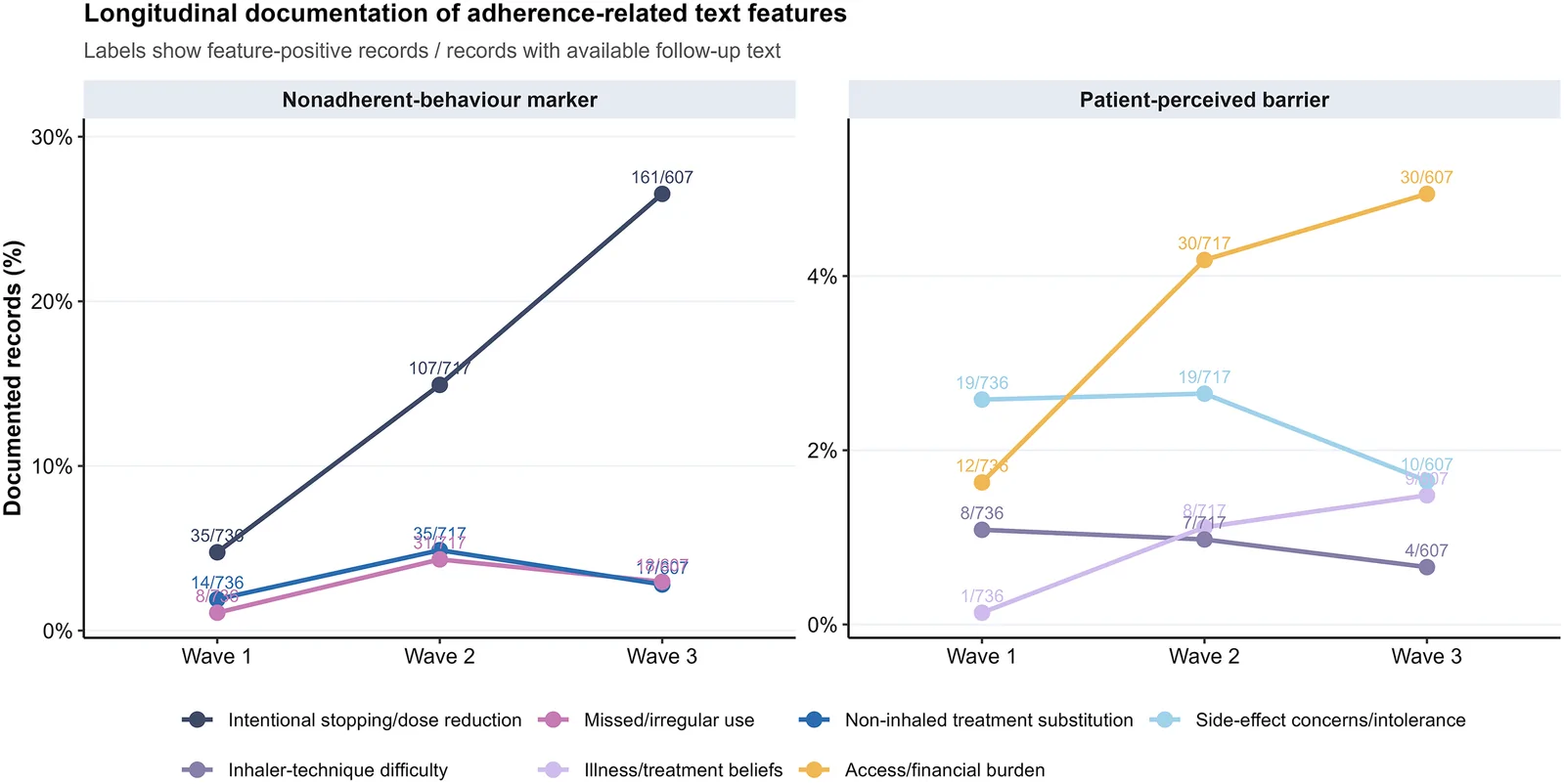
Documented frequencies of seven adherence-related text features across three follow-up waves.

### 3.3 Primary analysis: contemporaneous GEE associations between adherence-related features and high adherence

Both GEE models included 2,031 evaluable observations from 767 patients. In Model 1, after adjustment for age, sex, wave, and the other patient-perceived barriers, lower contemporaneous odds of high adherence were observed when access or financial barriers (OR 0.28, 95% CI 0.16–0.49; P<0.001), illness- or treatment-related beliefs (OR 0.19, 95% CI 0.06–0.63; P = 0.007), or side-effect concerns or poor tolerability (OR 0.43, 95% CI 0.19–0.96; P = 0.040) were documented. The estimate for inhaler device-related difficulty was imprecise and its confidence interval included 1 (OR 0.55, 95% CI 0.14–2.17; P = 0.389).

Model 2 additionally included intentional discontinuation or dose reduction, forgetfulness or irregular use, and alternative-therapy substitution. Each was co-documented with lower odds of high adherence, as were access or financial barriers, illness- or treatment-related beliefs, and side-effect concerns or poor tolerability (**Table 4**). Because the three added features overlap with the operational definition or documented context of low adherence, their coefficients describe same-record coding relationships rather than etiologic effects. Odds of high adherence were lower at Waves 2 and 3 than at Wave 1 in both models.

**Table 4:** Contemporaneous GEE results from the four-barrier and extended seven-feature models.

Variable	Model 1 OR (95% CI)	P value	Model 2 OR (95% CI)	P value
Intentional discontinuation or dose reduction	–	–	0.07 (0.05–0.10)	<0.001
Forgetfulness or irregular use	–	–	0.12 (0.06–0.25)	<0.001
Alternative-therapy substitution	–	–	0.37 (0.19–0.75)	0.006
Side-effect concerns or poor tolerability	0.43 (0.19–0.96)	0.040	0.43 (0.20–0.92)	0.028
Inhaler device-related difficulty	0.55 (0.14–2.17)	0.389	0.43 (0.12–1.57)	0.201
Illness- or treatment-related beliefs	0.19 (0.06–0.63)	0.007	0.19 (0.05–0.73)	0.016
Access or financial barriers	0.28 (0.16–0.49)	<0.001	0.27 (0.16–0.45)	<0.001
Wave 2 vs Wave 1	0.17 (0.12–0.23)	<0.001	0.22 (0.16–0.30)	<0.001
Wave 3 vs Wave 1	0.05 (0.03–0.07)	<0.001	0.06 (0.04–0.09)	<0.001
Age, per 1-SD increase	1.15 (1.01–1.30)	0.033	1.15 (1.00–1.33)	0.054
Male vs female	1.06 (0.81–1.38)	0.682	0.86 (0.64–1.15)	0.311

The outcome was contemporaneous high adherence. Both models included 2,031 observations from 767 patients and adjusted for age, sex, and wave. Model 1 included four prespecified patient-perceived barriers; Model 2 included all seven text features. Behavioral and treatment-substitution features partly overlapped with the outcome definition and were included only for exploratory co-documented analysis.

### 3.4 Adjacent-wave transitions in adherence and text features

Among 663 patients with evaluable adherence at both Waves 1 and 2, 475/628 (75.6%) with high adherence at Wave 1 remained high and 153/628 (24.4%) transitioned to low adherence. Of 35 patients with low adherence at Wave 1, 14 (40.0%) transitioned to high adherence and 21 (60.0%) remained low. Among 543 patients evaluable at Waves 2 and 3, 251/442 (56.8%) with high adherence at Wave 2 remained high and 191/442 (43.2%) transitioned to low adherence. Of 101 patients with low adherence at Wave 2, 18 (17.8%) transitioned to high adherence and 83 (82.2%) remained low (**Figure 2B**; **Table 3**; **Supplementary Figure S2**).

Across adjacent waves, text features were often newly documented or no longer documented, whereas persistent documented was less common (**Supplementary Figure S5**). These labels describe changes in recorded text only. Absence of later documented does not establish resolution of the underlying barrier.

Note: Percentages use the corresponding adherence state at the preceding wave as the denominator.

### 3.5 Subgroup analyses

In exploratory stratified analyses, access or financial barriers had estimates below 1 in both age groups and in women and men (**Table 5**). The nominal interaction P value was 0.012 for age and access or financial barriers. For sex and inhaler device-related difficulty, the nominal interaction P value was 0.029. Other estimates were imprecise, and several strata contained few positive records. Because these analyses involved multiple comparisons and sparse cells, they were treated as hypothesis generating and did not support firm effect-modification conclusions.

**Table 5:** Age- and sex-stratified analyses of the four patient-perceived barriers.

Stratification variable	Barrier	Subgroup	OR (95% CI)	P value	P for interaction
Age	Side-effect concerns or poor tolerability	<60	0.42 (0.16–1.06)	0.067	0.615
	Side-effect concerns or poor tolerability	≥60	0.50 (0.10–2.40)	0.386	
	Inhaler device-related difficulty	<60	0.66 (0.07–6.03)	0.710	0.879
	Inhaler device-related difficulty	≥60	0.49 (0.09–2.66)	0.411	
	Illness- or treatment-related beliefs	<60	0.15 (0.03–0.77)	0.023	0.517
	Illness- or treatment-related beliefs	≥60	0.28 (0.05–1.72)	0.169	
	Access or financial barriers	<60	0.33 (0.19–0.60)	<0.001	0.012
	Access or financial barriers	≥60	0.05 (0.02–0.16)	<0.001	
Sex	Side-effect concerns or poor tolerability	Female	0.38 (0.14–0.98)	0.045	0.336
	Side-effect concerns or poor tolerability	Male	0.75 (0.23–2.42)	0.627	
	Inhaler device-related difficulty	Female	0.78 (0.20–3.12)	0.729	0.029
	Inhaler device-related difficulty	Male	0.04 (0.00–0.31)	0.002	
	Illness- or treatment-related beliefs	Female	0.25 (0.07–0.87)	0.029	0.643
	Illness- or treatment-related beliefs	Male	0.12 (0.01–1.90)	0.133	
	Access or financial barriers	Female	0.34 (0.17–0.71)	0.004	0.475
	Access or financial barriers	Male	0.21 (0.09–0.51)	<0.001	

### 3.6 Sensitivity analyses

Wave-specific Firth models included 732, 697, and 602 adherence-evaluable records at Waves 1, 2, and 3, respectively (**Supplementary Table S11**). At Wave 1, side-effect concerns or poor tolerability (OR 0.09, 95% CI 0.03–0.28) and inhaler device-related difficulty (OR 0.10, 95% CI 0.02–0.61) were associated with lower odds of high adherence; only the former showed evidence of variation across waves (P for interaction <0.001 and 0.209, respectively). Illness- or treatment-related beliefs were associated with adherence at Wave 3 (OR 0.18, 95% CI 0.02–0.81), although the interaction was not estimable because of sparse Wave 1 records. Access or financial barriers showed ORs below 1 across all waves (range, 0.19–0.35), with no evidence of variation over time (P for interaction = 0.904).

The complete-case GEE included 511 participants and 1,533 observations (**Table 6**). Associations remained below 1 for access or financial barriers (OR 0.24, 95% CI 0.14–0.42; P<0.001) and illness- or treatment-related beliefs (OR 0.07, 95% CI 0.02–0.37; P = 0.001), whereas the confidence intervals for side-effect concerns or poor tolerability and inhaler device-related difficulty included 1.

**Table 6:** Sensitivity analyses.

	Wave 3 Firth regression		Complete-case GEE	
Variable	OR (95% CI)	P value	OR (95% CI)	P value
Side-effect concerns or poor tolerability	1.04 (0.30–3.54)	0.954	0.56 (0.25–1.26)	0.162
Inhaler device-related difficulty	0.72 (0.06–5.94)	0.756	0.90 (0.23–3.56)	0.878
Illness- or treatment-related beliefs	0.18 (0.02–0.81)	0.024	0.07 (0.02–0.37)	0.001
Access or financial barriers	0.35 (0.14–0.77)	0.009	0.24 (0.14–0.42)	<0.001
Age, per 1-SD increase	1.19 (1.01–1.40)	0.035	1.17 (1.04–1.33)	0.010
Male vs female	0.93 (0.67–1.29)	0.653	0.89 (0.69–1.14)	0.365

### 3.7 Missingness, treatment-regimen, and respondent-source analyses

Adherence was unevaluable in 35, 70, and 165 records at Waves 1, 2, and 3, respectively. Of 767 participants, 511 (66.62%) were evaluable at all three waves, while 256 had temporary, intermittent, or terminal missingness. Age and sex did not differ by evaluability. Relative to Wave 1, evaluability was lower at Wave 2 (OR 0.48, 95% CI 0.31–0.73) and Wave 3 (OR 0.17, 95% CI 0.12–0.26; both P<0.001). Prior adherence did not differ by Wave 2 evaluability but was lower among participants subsequently unevaluable at Wave 3 (47.40% vs. 81.40%; P<0.001) (**Supplementary Tables S2**–**S6**).

All participants received twice-daily Symbicort at baseline (160 strength, n=331; 320 strength, n=436). Adjustment for baseline strength yielded estimates similar to the primary analysis for side-effect concerns or poor tolerability (OR 0.43, 95% CI 0.19–0.96), illness- or treatment-related beliefs (OR 0.20, 95% CI 0.06–0.66), and access or financial barriers (OR 0.29, 95% CI 0.16–0.50). Baseline strength was not statistically associated with adherence (OR 1.24, 95% CI 0.95–1.61; P = 0.112) (**Supplementary Tables S7**, **S10**).

The respondent-source audit identified 1,977 patient-reported and 83 caregiver-reported records; 241 records had no narrative note, and no explicit joint interviews were identified. High adherence was 73.43% for patient-reported and 70.51% for caregiver-reported records. Caregiver versus patient reporting was not statistically associated with adherence (OR 0.73, 95% CI 0.41–1.28; P = 0.270), with similar results under an independence structure. In the patient-reported-only analysis, estimates remained below 1 for access or financial barriers (OR 0.30, 95% CI 0.17–0.52), illness- or treatment-related beliefs (OR 0.19, 95% CI 0.06–0.63), and side-effect concerns or poor tolerability (OR 0.42, 95% CI 0.19–0.96) (**Supplementary Tables S8**, **S9**).

## 4 Discussion

This study used three monthly telephone follow-ups from 767 patients newly initiating budesonide/formoterol for CVA. Among evaluable records, high adherence was documented less often at later waves, while intentional discontinuation or dose reduction was documented more often. In the primary four-barrier model, access or financial barriers, illness- or treatment-related beliefs, and side-effect concerns or poor tolerability were associated with lower contemporaneous odds of high adherence. Similar associations for access or financial barriers and illness- or treatment-related beliefs were observed in the complete-case and Wave 3 Firth analyses. These findings are descriptive and associational, not causal or predictive.

Among the patient-perceived barriers, access or financial barriers showed the most consistent association with adherence. Estimates remained below 1 in the primary, extended, complete-case, Wave 3 Firth, baseline-strength-adjusted, and patient-reported-only analyses. This consistency suggests that access-related difficulties are relevant topics to assess when low adherence is reported, but it does not establish that they caused the observed adherence status. The composite feature may encompass medication cost, drug availability, prescription renewal, and travel burden (17, 18). Because these mechanisms were not consistently distinguished in the follow-up notes, their individual contributions could not be evaluated.

Illness- or treatment-related beliefs were also associated with lower odds of high adherence in the primary, complete-case, and Wave 3 analyses. By contrast, the association for side-effect concerns or poor tolerability was less consistent across sensitivity analyses and estimates for inhaler device-related difficulty remained imprecise. These findings should be interpreted in light of the small numbers of some feature-positive records and simultaneous adjustment for correlated barriers. Moreover, inhaler technique was identified from telephone follow-up notes rather than standardized direct assessment, so undocumented technique errors may have been missed. Previous asthma studies have linked treatment beliefs, perceived necessity, and treatment concerns to adherence (19, 20). Evidence from COPD and other chronic diseases provides additional context, although it may not be directly generalizable to CVA (21, 22).

Intentional discontinuation or dose reduction increased from 4.8% of analyzable Wave 1 records to 26.5% at Wave 3. The reason for this pattern could not be determined because standardized longitudinal measures of symptom control and complete information on clinician-directed treatment changes were unavailable. Furthermore, intentional discontinuation or dose reduction, forgetfulness or irregular use, and alternative-therapy substitution overlap with the operational definition or documented context of low adherence. Their coefficients in the extended model therefore primarily reflect contemporaneous co-documentation rather than independent causal effects.

The GEE models estimated population-averaged associations between text features and adherence documented in the same follow-up record. Temporal ordering was therefore not established, and the findings may be influenced by common-source measurement and documentation practices. In addition, adherence was increasingly unevaluable across waves, and Wave 2 adherence differed between participants with and without an evaluable Wave 3 outcome. Consequently, wave-specific proportions may reflect both within-person changes among observed records and changes in the composition of the evaluable sample. Exploratory wave-specific analyses suggested that some associations may also vary over time. Side-effect concerns or poor tolerability showed the strongest association at Wave 1, with evidence of heterogeneity across waves. Inhaler device-related difficulty was also associated with adherence at Wave 1, although its interaction with wave was not statistically significant. Illness- or treatment-related beliefs were associated with adherence at Wave 3, but sparse Wave 1 records prevented reliable assessment of temporal heterogeneity. Access or financial barriers showed associations in the same direction across all three waves, with no statistical evidence of variation by wave. These temporal patterns are hypothesis-generating but suggest that adherence barriers may need to be reassessed during follow-up rather than evaluated only at treatment initiation.

The findings may help inform the content of future standardized adherence assessments. Follow-up could address affordability and medication access, treatment beliefs, adverse effects, inhaler use, missed doses, and patient-initiated treatment changes. However, this study did not evaluate an intervention, and no specific adherence-support strategy can be inferred to be effective. Educational and adherence interventions have shown heterogeneous effects in asthma (23). Digital and eHealth interventions have likewise produced variable results (24–26), and evidence for electronic monitoring remains context dependent (27, 28). CVA-specific interventions therefore require prospective evaluation.

This study has several limitations. First, the single-center observational design limits generalizability and precludes causal interpretation. Second, adherence was a qualitative record-based classification derived from patient or caregiver reports. It had no fixed dose-percentage threshold and was not validated against electronic monitoring, refill records, or medication counts. Third, adherence and text features were derived from the same notes. Failure to document a feature does not establish its absence. Fourth, missingness increased across waves and later evaluability was associated with prior adherence, so selection may affect wave-specific comparisons. Fifth, respondent source was classified retrospectively from explicit textual cues and was not manually validated. Caregiver-source records were few, and the analyses do not establish equivalence with patient reports. Sixth, baseline Symbicort strength did not capture inhalations per administration or all between-wave regimen changes. Standardized disease-control measures, education, health literacy, income, residence, and complete clinician-directed changes were unavailable, leaving residual confounding. Seventh, CVA diagnoses could not be re-adjudicated using one uniform test-based algorithm. Eighth, several barrier and subgroup estimates were based on sparse positive records. Finally, the rule-based NLP system was internally validated in 200 records, but external validation is required in other settings (29, 30).

## 5 Conclusions

In this single-center inception cohort of patients with CVA initiating twice-daily budesonide/formoterol, high adherence was documented less frequently at later follow-up waves, whereas patient-initiated discontinuation or dose reduction was documented more frequently. Access or financial barriers showed the most consistent contemporaneous association with lower odds of high adherence, while associations with illness- or treatment-related beliefs and side-effect concerns were less consistent across analyses. Exploratory findings suggested that the relevance of some barriers may vary during follow-up, supporting repeated rather than one-time assessment. Given the observational, record-based design, these findings require confirmation in prospective studies using standardized adherence and barrier measurements.

## Data Availability

The de-identified data supporting the conclusions of this article may be made available by the corresponding author upon reasonable request, subject to ethical and institutional requirements.
