# Supplementary material for: Inhaled treatment adherence patterns and related barriers across three monthly follow-up waves among patients with cough variant asthma: a clinical natural language processing study
Source: Front Immunol. 2026 Oct 5;17:1966303. doi: 10.3389/fimmu.2026.1966303 (PMC13677862; doi:10.3389/fimmu.2026.1966303)
Supplement: Supplementary file 1 [file Supplementaryfile1.docx]

**Supplementary materials**

**Supplementary Figure S1.** Rule-based clinical natural language processing workflow for identifying adherence-related text features from free-text follow-up records. Free-text records were normalized and cleaned, followed by removal of clinician-directed or non-patient-reported content using predefined trimming rules. The retained text was screened using positive pattern matching and negation or exclusion rules. Records meeting a positive pattern without a corresponding negation or exclusion were classified as containing the relevant adherence-related feature; otherwise, the feature was classified as not identified. Seven binary feature indicators were summarized. NLP, natural language processing.

**Supplementary Figure S2.** Adjacent-wave adherence-state transitions. Transitions in adherence status from Wave 1 to Wave 2 and from Wave 2 to Wave 3.

**Supplementary Figure S3.** Adherence-related text features documented at any wave. Proportions of participants with each of the seven adherence-related text features documented during at least one follow-up wave.

**Supplementary Figure S4.** Wave-specific documented frequencies of adherence-related text features. The heatmap displays the documented frequencies of each text feature across the three follow-up waves.

**Supplementary Figure S5.** Adjacent-wave dynamics of the seven text features. Changes in documented status between adjacent waves, including continued absence, newly documented, persistent, and no longer documented states.

**Supplementary Figure S6.** Pairwise phi coefficients among seven text features. The heatmap presents pairwise phi coefficients between binary text features; positive and negative values indicate co-occurrence and inverse associations, respectively.

**Supplementary Appendix S1. Domains Covered During Routine Semi-structured Telephone Follow-up**

Telephone follow-up was conducted using routine semi-structured clinical prompts rather than a validated adherence questionnaire. The wording and order of questions could vary according to the patient’s or caregiver’s responses and the clinical context. Not every domain was necessarily documented at every contact. Information obtained during the contact was summarized in a narrative clinical note.

| **Domain** | **Core prompts used during follow-up** | **Information documented** |
| --- | --- | --- |
| **Respondent identity** | Was the information provided by the patient or by a caregiver? If reported by a caregiver, what was the relationship to the patient? | Patient- or caregiver-reported source when explicitly documented. |
| **Current respiratory symptoms** | Is cough still present? Has it improved, worsened, or remained unchanged? How frequent or severe is it? Are sputum, chest tightness, dyspnea, or nocturnal symptoms present? | Descriptive symptom status and changes since the previous contact. These observations were not collected using a standardized symptom-control scale. |
| **Current inhaled-treatment implementation** | Are you currently using the prescribed inhaler? How often are you actually using it? When was it last used? Is medication still available? | Current use, actual reported frequency, interruption of treatment, and remaining medication when mentioned. |
| **Missed or irregular use** | Have any doses been missed or used irregularly? Was the irregular use occasional or recurrent? | Reported missed doses, irregular use, forgetfulness, disrupted routine, travel, or other practical reasons. |
| **Dose reduction or discontinuation** | Have you reduced the dose or frequency, temporarily interrupted treatment, or stopped using the inhaler? When did this occur? Was the change made by you or directed by a clinician? | Patient-initiated dose reduction, interruption, or discontinuation, distinguished from clinician-directed treatment changes whenever this information was available. |
| **Reasons for treatment modification** | Why was the inhaler missed, reduced, or stopped? Was this related to symptom change, perceived lack of benefit, concern about adverse effects, difficulty using the device, medication availability, cost, or another reason? | Patient-reported reasons for nonadherent implementation. No reason was inferred when the narrative information was insufficient. |
| **Inhaler-device use** | Are you able to use the inhaler? Is there any difficulty operating the device, coordinating inhalation, identifying whether a dose was delivered, or understanding the dose indicator? | Reported device-use ability, specific difficulties, telephone guidance provided, and recommendation for in-person technique reassessment when required. |
| **Adverse effects and tolerability** | Have you experienced discomfort or concerns that affected inhaler use, such as throat or oral discomfort, hoarseness, or concerns about corticosteroid-related effects? Do you rinse your mouth after inhalation? | Reported adverse-effect concerns, tolerability problems, and whether these influenced treatment implementation. |
| **Medication access and prescription renewal** | Is the inhaler running out or already finished? Can you obtain a refill or return for follow-up? Are cost, travel, availability, or prescription renewal creating difficulty? | Medication availability, refill or prescription-renewal difficulties, planned clinic attendance, and access-related treatment interruption. |
| **Alternative or concomitant treatments** | Are you using traditional Chinese medicine, oral medication, home remedies, health products, or treatment from another institution? Has this replaced, delayed, or reduced use of the prescribed inhaler? | Alternative or concomitant treatment and its documented effect on prescribed inhaled-treatment implementation. |
| **Clinician-directed changes and follow-up plan** | Has a clinician advised changing, stepping down, or stopping treatment? Is further outpatient review or prescription renewal planned? | Clinician-directed treatment adjustments, advice provided during follow-up, and the documented follow-up or refill plan. |

**Note:** The guide was used to support routine clinical follow-up and was not administered as a fixed-item or validated adherence questionnaire. Dose counts and prescribed-dose denominators were not consistently documented; therefore, a numerical adherence threshold such as ≥80% of prescribed doses was not applied. Symptom descriptions obtained during follow-up were not treated as standardized longitudinal disease-control measurements

**Supplementary Table S1. Operational definitions for adherence classification and adherence-related text features**

Panel A. Operational definitions of the seven non-mutually exclusive adherence-related text features

| **Text feature** | **Operational definition** | **Representative positive expressions** | **Negation or exclusion rules** |
| --- | --- | --- | --- |
| Intentional discontinuation or dose reduction | Patient-initiated discontinuation, temporary interruption, dose reduction, or reduced frequency without clinician instruction | “stopped using it,” “used less,” “reduced to once daily,” “only used it when symptoms occurred,” “stopped after feeling better” | Clinician-directed discontinuation, dose reduction, treatment completion, or documented step-down according to medical advice |
| Forgetfulness or irregular use | Unintentional missed doses or inconsistent medication use caused by forgetting, disrupted routines, travel, or inconvenience | “forgot to use it,” “occasionally missed a dose,” “used it irregularly,” “did not bring it when travelling,” “sometimes remembered and sometimes forgot” | Deliberate non-use, clinician-approved intermittent treatment, or isolated delayed administration without missed treatment |
| Side-effect concerns or poor tolerability | Concern about adverse effects or actual discomfort attributed to inhaled treatment that affected willingness or ability to continue treatment | “worried about steroid side effects,” “afraid of dependence,” “hoarseness after use,” “felt uncomfortable after inhalation,” “stopped because of side effects” | Adverse effects explicitly denied, general safety questions without treatment impact, or symptoms clearly unrelated to the medication |
| Inhaler device-related difficulty | Difficulty understanding, operating, coordinating, or correctly using the inhalation device | “did not know how to use the device,” “could not press and inhale at the same time,” “unsure whether the dose was inhaled,” “the device was difficult to operate” | Correct technique documented, difficulty resolved after instruction, or problems unrelated to device operation |
| Illness- or treatment-related beliefs | Misunderstanding or limited acceptance of the diagnosis, chronic nature of the condition, or need for maintenance therapy | “did not think the condition required long-term treatment,” “used medicine only when coughing,” “thought treatment was unnecessary after symptoms improved,” “did not believe inhaled therapy was needed” | Correct understanding of maintenance treatment, continued treatment despite symptom relief, or neutral questions without evidence of misconception |
| Access or financial barriers | Medication interruption or difficulty obtaining treatment because of cost, prescription renewal, availability, distance, or service accessibility | “the medicine was too expensive,” “could not obtain a refill,” “the pharmacy did not have it,” “it was inconvenient to return for a prescription,” “stopped because the medication ran out” | Treatment interruption for reasons unrelated to access, successfully obtaining medication without interruption, or general comments about cost without an effect on treatment |
| Alternative-therapy substitution | Replacement, postponement, or reduction of prescribed inhaled treatment because of preference for traditional medicine, home remedies, health products, or other alternative approaches | “preferred traditional Chinese medicine,” “used herbal medicine instead,” “stopped inhalation after trying a home remedy,” “wanted to manage the cough without inhaled medication” | Alternative treatment used together with prescribed inhalation without reducing adherence, or alternative therapy recommended as part of clinician-directed care |

**Panel B. Adherence outcome classification**

| **Classification** | **Operational definition** | **Representative documentation** | **Exclusions and special rules** |
| --- | --- | --- | --- |
| **High adherence** | The record contained sufficient information indicating implementation of the prescribed inhaled therapy during the preceding month, without explicit documentation of recurrent or irregular missed use, patient-initiated dose reduction, or patient-initiated discontinuation. | Regular use as prescribed; continued inhaled treatment without reported interruption; no recurrent or irregular missed use or self-directed treatment adjustment documented. | Clinician-directed dose reduction, discontinuation, or regimen adjustment was not classified as low adherence. Absence of a barrier term alone was insufficient to classify adherence as high. |
| **Low adherence** | The record explicitly documented recurrent or irregular missed use, patient-initiated dose reduction, patient-initiated discontinuation, or replacement/postponement of the prescribed inhaled therapy with an alternative treatment during the preceding month. | Irregular inhaler use; repeated missed doses; self-reduction of dose or frequency; self-discontinuation; replacement of inhaled treatment with traditional medicine, supplements, or another non-prescribed therapy. | Treatment changes explicitly directed by a clinician were excluded. Symptoms or concerns without documented effects on treatment implementation were not sufficient for classification as low adherence. |
| **Unevaluable adherence** | The available record did not contain sufficient information to determine how the prescribed inhaled therapy had been implemented during the preceding month. | No narrative note; medication mentioned without information on actual use; ambiguous or incomplete documentation from which regularity of use could not be determined. | Unevaluable records were treated as missing outcomes for the corresponding wave and were not automatically classified as either high or low adherence. |

**Note:** Adherence was classified qualitatively from patient- or caregiver-reported treatment implementation documented in routine telephone follow-up notes. Because dose counts and prescribed-dose denominators were not consistently available, a numerical threshold such as ≥80% of prescribed doses was not applied. Classification was based on the preceding month. Clinician-directed treatment adjustments were excluded from low-adherence behaviors.

**Supplementary Table S2. Age and sex by adherence evaluability at each wave**

| **Wave** | **Variable** | **Evaluable** | **Unevaluable** | **P value** |
| --- | --- | --- | --- | --- |
| Wave 1 | Age, years | 46.0 ± 13.9 (n=732) | 49.8 ± 13.3 (n=35) | 0.111 |
| Wave 1 | Female sex | 449/732 (61.3%) | 19/35 (54.3%) | 0.403 |
| Wave 2 | Age, years | 46.2 ± 14.0 (n=697) | 45.7 ± 13.3 (n=70) | 0.725 |
| Wave 2 | Female sex | 419/697 (60.1%) | 49/70 (70.0%) | 0.106 |
| Wave 3 | Age, years | 46.6 ± 14.0 (n=602) | 44.4 ± 13.5(n=165) | 0.067 |
| Wave 3 | Female sex | 359/602 (59.6%) | 109/165 (66.1%) | 0.134 |

Age was compared using the Wilcoxon rank-sum test and sex using the chi-square or Fisher exact test, as appropriate. Counts are based on the revised cohort; P values are descriptive.

**Supplementary Table S3. Participant characteristics by complete three-wave adherence evaluability**

| **Variable** | **All three waves evaluable (n=511)** | **Any wave unevaluable (n=256)** | **P value** |
| --- | --- | --- | --- |
| Age, years | 46.5 ± 14.1 | 45.5 ± 13.5 | 0.351 |
| Female sex | 300/511 (58.7%) | 168/256 (65.6%) | 0.076 |

Complete evaluability required an assessable adherence outcome at all three waves.

**Supplementary Table S4. Three-wave adherence-evaluability patterns and grouped missingness types**

| **Pattern/type** | **Definition** | **N（%）** |
| --- | --- | --- |
| OOO | Evaluable at all three waves | 511（66.62） |
| MOO | Wave 1 unevaluable; Waves 2–3 evaluable | 32(4.17) |
| OMO | Wave 2 unevaluable; Waves 1 and 3 evaluable | 58(7.56%) |
| MMO | Waves 1–2 unevaluable; Wave 3 evaluable | 1(0.13) |
| MOM | Waves 1 and 3 unevaluable; Wave 2 evaluable | 2(0.26) |
| OMM | Wave 1 evaluable; Waves 2–3 unevaluable | 11(1.43) |
| OOM | Waves 1–2 evaluable; Wave 3 unevaluable | 152(19.82) |
| Temporary missingness | MOO or OMO | 90(11.73) |
| Intermittent/recurrent missingness | MMO or MOM | 3(0.39) |
| Persistent/terminal missingness | OMM or OOM | 163(21.25) |
| One unevaluable wave | Any pattern with exactly one M | 242(31.55) |
| Two unevaluable waves | Any pattern with exactly two M | 14(1.83) |

Note: O=evaluable adherence; M=missing/unevaluable adherence. No participant in the final analytic cohort had all three waves unevaluable.

**Supplementary Table S5. Prior-wave high adherence according to subsequent-wave evaluability**

| **Eligible prior-wave group** | **Next wave evaluable** | **Next wave unevaluable** | **P value** |
| --- | --- | --- | --- |
| Wave 1 adherence evaluable (n=732): Wave 1 high adherence | 628/663 (94.72%) | 63/69 (91.30%) | 0.265 |
| Wave 2 adherence evaluable (n=697): Wave 2 high adherence | 442/543 (81.40%) | 73/154 (47.40%) | <0.001 |

Note: The first comparison relates Wave 1 adherence to Wave 2 evaluability; the second relates Wave 2 adherence to Wave 3 evaluability. P values are descriptive and do not establish a missing-data mechanism.

**Supplementary Table S6. Logistic GEE for adherence evaluability**

| **Variable** | **OR (95%CI)** | **P value** |
| --- | --- | --- |
| Age | 1.07 (0.94–1.21) | 0.299 |
| Male sex | 1.27 (0.98–1.65) | 0.073 |
| Wave 2 vs Wave 1 | 0.48 (0.31–0.73) | <0.001 |
| Wave 3 vs Wave 1 | 0.17 (0.12–0.26) | <0.001 |

Note: Outcome: adherence evaluable at the scheduled wave. The exchangeable logistic GEE included 2,301 scheduled observations from 767 participants and used patient-clustered robust standard errors.

**Supplementary Table S7. Baseline maintenance inhaler prescription audit**

| **Prescription characteristic** | **N(%)** |
| --- | --- |
| Symbicort explicitly documented | 767 |
| Symbicort 160 strength | 331(43.15) |
| Symbicort 320 strength | 436(56.84) |

Note: All participants were newly initiated on the documented maintenance inhaler at cohort entry. The number of inhalations per administration and between-wave regimen changes were not consistently available. Twice-daily prescription: 767 .

**Supplementary Table S8A. Post hoc rule-based respondent-source audit**

| **Source category** | **Overall** | **Wave 1** | **Wave 2** | **Wave 3** |
| --- | --- | --- | --- | --- |
| Patient source | 1977 | 706 | 687 | 584 |
| Caregiver source | 83 | 30 | 30 | 23 |
| No narrative note | 241 | 31 | 50 | 160 |
| Total | 2301 | 767 | 767 | 767 |

Note: Source categories were derived retrospectively from explicit narrative cues and were not manually validated. A mixed multi-source note does not necessarily represent joint participation in the same telephone call.

**Supplementary Table S8B. High adherence by respondent source among evaluable records**

| **Source** | **Overall** | **Wave 1** | **Wave 2** | **Wave 3** |
| --- | --- | --- | --- | --- |
| Patient source | 1434/1953(73.43) | 663/702(94.44) | 496/672(73.81) | 275/579(47.50) |
| Caregiver source | 55/78(70.51) | 28/30(93.33) | 19/25(76.00) | 8/23(34.78) |

Note: Percentages are descriptive. Differences in case mix, small caregiver-source counts, and retrospective source classification preclude an equivalence conclusion.

**Supplementary Table S9A. Respondent-source GEE among patient- and caregiver-source records**

| **Working correlation** | **Comparison** | **OR (95%CI)** | **P value** |
| --- | --- | --- | --- |
| Independence | Caregiver vs patient | 0.70 (0.39–1.25) | 0.230 |
| Exchangeable | Caregiver vs patient | 0.73 (0.41–1.28) | 0.270 |

Note: Outcome: contemporaneous high adherence. Models adjusted for age, sex, and wave and used patient-clustered robust standard errors. The confidence intervals are wide and do not demonstrate equivalence.

**Supplementary Table S9B. Patient-source-only primary four-barrier GEE**

| **Variable** | **OR(95% CI)** | **P value** |
| --- | --- | --- |
| Side-effect concerns/poor tolerability | 0.42 (0.19–0.96) | 0.040 |
| Device-related difficulty | 0.64 (0.13–3.07) | 0.577 |
| Illness/treatment beliefs | 0.19 (0.06–0.63) | 0.006 |
| Access or financial barriers | 0.30 (0.17–0.52) | <0.001 |
| Wave 2 vs Wave 1 | 0.16 (0.12–0.23) | <0.001 |
| Wave 3 vs Wave 1 | 0.05 (0.03–0.07) | <0.001 |
| Age, per 1-SD increase | 1.16 (1.02–1.33) | 0.029 |
| Male sex | 1.04 (0.80–1.36) | 0.770 |

**Supplementary Table S10. Primary four-barrier GEE additionally adjusted for baseline Symbicort strength**

| **Variable** | **OR(95% CI)** | **P value** |
| --- | --- | --- |
| Side-effect concerns or poor tolerability | 0.43 (0.19–0.96) | 0.039 |
| Inhaler device-related difficulty | 0.53 (0.13–2.12) | 0.373 |
| Illness- or treatment-related beliefs | 0.20 (0.06–0.66) | 0.009 |
| Access or financial barriers | 0.29 (0.16–0.50) | <0.001 |
| Symbicort 320 vs 160 | 1.24 (0.95–1.61) | 0.112 |
| Wave 2 vs Wave 1 | 0.17 (0.12–0.23) | <0.001 |
| Wave 3 vs Wave 1 | 0.05 (0.03–0.07) | <0.001 |
| Age, per 1-SD increase | 1.14 (1.00–1.29) | 0.044 |
| Male sex | 1.06 (0.81–1.38) | 0.665 |

**Supplementary Table S11. Wave-specific associations between patient-perceived barriers and high adherence**

| **Patient-perceived barrier** | **Wave 1 (n=732)** | | **Wave 2 (n=697)** | | **Wave 3 (n=602)** | | **P for interaction** |
| --- | --- | --- | --- | --- | --- | --- | --- |
|  | **OR (95% CI)** | **P value** | **OR (95% CI)** | **P value** | **OR (95% CI)** | **P value** |  |
| Side-effect concerns or poor tolerability | 0.09 (0.03–0.28) | <0.001 | 0.62 (0.24–1.74) | 0.346 | 1.04 (0.30–3.54) | 0.954 | <0.001 |
| Inhaler device-related difficulty | 0.10 (0.02–0.61) | 0.016 | 1.18 (0.23–11.66) | 0.854 | 0.72 (0.06–5.94) | 0.756 | 0.209 |
| Illness- or treatment-related beliefs | 0.20 (0.01–4.27) | 0.303 | 0.45 (0.09–2.72) | 0.352 | 0.18 (0.02–0.81) | 0.024 | NA |
| Access or financial barriers | 0.19 (0.05–1.04) | 0.055 | 0.29 (0.14–0.62) | 0.002 | 0.35 (0.14–0.77) | 0.009 | 0.904 |

Note: Wave-specific estimates were obtained using multivariable Firth penalized logistic regression. P values for interaction were derived from barrier-by-wave interaction terms in the GEE. NA indicates that the overall interaction could not be reliably estimated because of sparse records. All wave-specific and interaction analyses were exploratory.
